# A Longitudinal Assessment of Sleep Timing, Circadian Phase, and Phase Angle of Entrainment across Human Adolescence

**DOI:** 10.1371/journal.pone.0112199

**Published:** 2014-11-07

**Authors:** Stephanie J. Crowley, Eliza Van Reen, Monique K. LeBourgeois, Christine Acebo, Leila Tarokh, Ronald Seifer, David H. Barker, Mary A. Carskadon

**Affiliations:** 1 Biological Rhythms Research Laboratory, Department of Behavioral Sciences, Rush University Medical Center, Chicago, IL, United States of America; 2 E.P. Bradley Hospital Sleep Research Laboratory, Department of Psychiatry and Human Behavior, Warren Alpert Medical School of Brown University, Providence, RI, United States of America; 3 Sleep and Development Laboratory, Department of Integrative Physiology, University of Colorado Boulder, Boulder, CO, United States of America; 4 The Bradley Hasbro Children's Research Center, Providence, RI, United States of America; 5 Institute of Pharmacology and Toxicology, University of Zurich, Zurich, Switzerland; 6 University Hospital of Child and Adolescent Psychiatry, University of Bern, Bern, Switzerland; 7 Centre for Sleep Research, University of South Australia, Adelaide, Australia; Oregon Health & Science University, United States of America

## Abstract

The aim of this descriptive analysis was to examine sleep timing, circadian phase, and phase angle of entrainment across adolescence in a longitudinal study design. Ninety-four adolescents participated; 38 (21 boys) were 9–10 years (“younger cohort”) and 56 (30 boys) were 15–16 years (“older cohort”) at the baseline assessment. Participants completed a baseline and then follow-up assessments approximately every six months for 2.5 years. At each assessment, participants wore a wrist actigraph for at least one week at home to measure self-selected sleep timing before salivary dim light melatonin onset (DLMO) phase – a marker of the circadian timing system – was measured in the laboratory. Weekday and weekend sleep onset and offset and weekend-weekday differences were derived from actigraphy. Phase angles were the time durations from DLMO to weekday sleep onset and offset times. Each cohort showed later sleep onset (weekend and weekday), later weekend sleep offset, and later DLMO with age. Weekday sleep offset shifted earlier with age in the younger cohort and later in the older cohort after age 17. Weekend-weekday sleep offset differences increased with age in the younger cohort and decreased in the older cohort after age 17. DLMO to sleep offset phase angle narrowed with age in the younger cohort and became broader in the older cohort. The older cohort had a wider sleep onset phase angle compared to the younger cohort; however, an age-related phase angle increase was seen in the younger cohort only. Individual differences were seen in these developmental trajectories. This descriptive study indicated that circadian phase and self-selected sleep delayed across adolescence, though school-day sleep offset advanced until no longer in high school, whereupon offset was later. Phase angle changes are described as an interaction of developmental changes in sleep regulation interacting with psychosocial factors (e.g., bedtime autonomy).

## Introduction

The transition through adolescence (the second decade) is often accompanied by a shift toward later timing of sleep/wake behavior. Survey studies from around the globe report later bedtimes on both school and non-school nights and later wake-up times on non-school or vacation mornings as youngsters age [1], [2], [3], [4], [5], [6], [7]. Cross-sectional and longitudinal studies using actigraphically-estimated sleep support these findings [8], [9]. A shift toward “eveningness” also emerges as youngsters age [10], and this shift appears to be linked to pubertal development [11], [12]. For American students, this delay of sleep behavior is often concurrent with the earliest school start times, reducing the opportunity for sleep on school nights for many. Cross-sectional and longitudinal studies of adolescent sleep length reflect this circumstance, showing a consistent age-related reduction of total sleep time [1], [9], [13], [14], [15], [16], [17], [18], [19]. Restricted weekday sleep during the school year is often compensated by over-sleeping on weekends, primarily through later wake-up times [1], [4], [20], [21].

Many studies reporting sleep/wake behavioral timing across adolescence have used cross-sectional comparisons of several age groups. While cross-sectional studies are a crucial step to understanding developmental differences, the findings can be misleading as this approach does not account for individual differences in developmental trajectories [22]. Longitudinal studies allow one to better understand developmental trajectories of behavior. Of the few studies reporting longitudinal patterns of adolescent sleep/wake timing, two examined young adolescents (∼10–13 years) [2], [9]. Laberge and colleagues reported an age-related delay in parent-reported bedtimes on school and weekend nights, and a delay in weekend wake-up time over four years in young Canadian adolescents. Sadeh and colleagues found a similar age-related delay in sleep onset time measured by actigraphy in a group of young adolescents (9.9 to 11.2 years at baseline) followed over 3 years, and this change in sleep timing predicted changes to self-assessed puberty ratings. Andrade and colleagues [20] reported a delay in self-reported weekend wake-up time (on average between 34 and 39 minutes) in Brazilian adolescents (12–16 years); however, measurements were taken over one year only. Weekday and weekend bedtime and weekday wake time did not change over this year. The last longitudinal study focused on the transition from high school to college [23]. Urner and colleagues measured actigraphically-estimated sleep/wake patterns in Swiss students while in high school (aged 17 to 19 years) and again 5 years later when in university. Bedtime, wake-up time, and mid-sleep time shifted on average 34, 52, and 44 minutes later on school days after the transition to college, but sleep timing did not change on non-school days. In summary, previous longitudinal studies focused on early [2], [9] or late adolescence [23] and the only longitudinal study that focused on a relatively wide age range, followed youngsters for one year only [20]. Thus, longitudinal assessments of sleep/wake timing using objective measures at more frequent intervals and spanning the second decade of life are needed to understand developmental trajectories of sleep/wake behavior during adolescence.

Delayed sleep timing during adolescence is partly driven by environmental factors that can displace sleep, such as part-time work, homework, television-watching, or other media use [24], [25], [26]; however, changes to the homeostatic sleep [27] and circadian timing systems [28], [29], particularly during pubertal development, can also explain delayed sleep/wake timing in these youngsters. Previous studies in adolescent humans [28], [29] and other young mammals [30] report a puberty-related delay of the circadian timing system. In the cross-sectional studies of Carskadon and colleagues [28], [29], for example, the melatonin rhythm was later in participants who were late- or post-pubertal compared to those who were pre- or early pubertal. Of note is that all of these youngsters kept the same sleep/wake (and thus dark/light) pattern for at least one week before circadian phase measurement, which indicates that changes to circadian timing were not driven by differences in sleep/wake patterns. Given a fixed sleep/wake (dark/light) schedule and differences in circadian timing, these data may also indicate developmental differences in the temporal alignment of sleep/wake behavior with the internal circadian clock. Such temporal alignment is referred to as the phase angle of entrainment, i.e., the time interval between an endogenous circadian marker (e.g., the dim light melatonin onset, or DLMO) and a recurring external cyclic event (e.g., sleep onset or sleep offset). Indeed, a previous cross-sectional analysis of adolescents reported an age-related difference in the interval from DLMO to bedtime, particularly during the school year, with an older group (13–16 years) showing a wider phase angle compared to a younger group (9–12 years) [31]. To date, no published studies have examined developmental changes to circadian physiology or the temporal alignment between the circadian system and sleep/wake behavior using a longitudinal design in adolescent humans.

The current study is a descriptive longitudinal evaluation of actigraphically-estimated weekday and weekend sleep onset and offset times, weekend-to-weekday differences in sleep onset and offset times, circadian phase (measured using salivary DLMO phase) and phase angles of entrainment in a younger adolescent cohort and an older adolescent cohort. We hypothesized that participants in both cohorts would show later DLMOs, sleep onsets on weekends and weekdays, and sleep offsets on weekends over time and that weekend sleep onset and sleep offset differences would increase with age. We did not have specific hypotheses for the developmental changes of phase angles of entrainment.

## Methods

### Participants

Two cohorts of adolescents living in the northeast United States (41° 49′ N, 71° 24′ W) participated in this study from 2002 to 2007: a younger cohort first assessed at age 9 or 10 years and an older cohort whose baseline assessment occurred at age 15 or 16 years. Participants were screened for the following exclusion criteria at the initial assessment using questionnaires completed by participants and a parent: chronic insufficient sleep concomitant with signs of excessive sleepiness, such as falling asleep inappropriately; more than 3-hour variation in self-reported sleep times across a week; personal or family history of a medical, psychiatric, or sleep disorder; current illness; use of prescription medications or over-the-counter medications known to affect sleep, alertness, or suppress melatonin; or physical or mental handicap. The Lifespan Institutional Review Board approved the study, and participants received payment for taking part in each assessment. A parent gave written informed consent, and participants co-signed to indicate their assent to participate in the study. Participants gave written informed consent if they were 18 years or older.

### Procedures

Procedures were the same for both cohorts, and assessments were scheduled to occur during the school year, excluding vacations and holidays and not within one week of transition to Daylight Saving Time. Participants were invited for a baseline assessment and for follow-up assessments approximately every six months for 2.5 years thereafter (total planned assessments  = 6). At each assessment, participants wore an actigraph on their non-dominant wrist (Mini-motionlogger, Ambulatory Monitoring, Inc., Ardsley, NY, USA) and kept a daily sleep diary in which they documented their sleep pattern. This monitoring occurred for at least one week before coming to the laboratory to measure the salivary dim light melatonin onset (DLMO) phase on a weekday evening. Participants were free to select their sleep times, and they were instructed to sleep at home alone and not remain awake all night. Activity data were collected in 1-minute epochs using a Zero-Crossing Mode (ZCM) and filter setting 18 (the manufacturer's setting for frequency bandpass of 2 to 3 Hz). Further verification of bedtimes and wake-up times were provided with twice-daily telephone calls to the laboratory's time-stamped answering machine, immediately before going to bed and immediately after waking.

Saliva collection occurred in the laboratory under dim light conditions (<40 lux) using untreated Salivettes (Starstedt, Germany) at 30-minute intervals. Participants were seated for five minutes before each of the twelve saliva samples collected beginning 5 hours before the participant's average bedtime (as calculated from daily telephone messages) and ending 30 minutes after average bedtime. If participants ingested anything other than water before the sample, they rinsed their mouths and brushed their teeth with water. Saliva samples were centrifuged, frozen, and later assayed for melatonin using radioimmunoassay (RIA) test kits (ALPCO, Windham, NH, USA). An individual's samples were analyzed in the same batch. The intra-assay coefficients of variation for low and high levels of salivary melatonin were 4.1% and 4.8%, respectively. The inter-assay coefficients of variation for low and high levels of salivary melatonin were 6.6% and 8.4%, respectively. The functional least detectable dose of the assay (minimum salivary melatonin concentration measured with an intra-assay coefficient of variation of less than 10%) was 0.9 pg/mL.

A physician examined participants at each assessment to determine pubertal status using the criteria of Tanner [32], a staging system based on secondary sexual characteristics with a range from stage 1 (i.e., child-like pre-pubertal) to 5 (post-pubertal). Classification presented here is for pubic hair growth [32]. Morningness/eveningness, a measure of when someone feels at their peak level of functioning, was also measured at each assessment using the Morningness questionnaire of Smith and colleagues [33]. Scores on this scale range from 10 to 56, with a lower score indicating eveningness. Alcohol and medication that would affect melatonin levels or sleep were prohibited throughout the assessment weeks. Caffeine and chocolate were prohibited after noon each day.

### Outcome measures

Actigraphic sleep data were analyzed using the Action-W2 software (version 2.3.20, Ambulatory Monitoring, Inc., Ardsley, NY, USA) to estimate sleep/wake using the validated “Sadeh” algorithm [34]. Each sleep episode was inspected within a scoring interval spanning 15-minutes before participants reported trying to fall asleep to 15-minutes after reported wake-up time on their daily sleep diary. The following variables were derived according to the procedures of Acebo and colleagues [35]: sleep onset time (the first minute of at least 3 consecutive minutes of sleep), sleep offset time (the last minute of at least 5 consecutive minutes of sleep before the end of the scoring interval), and sleep minutes (minutes of the sleep interval scored as sleep). If periods of low activity persisted outside the scoring interval, members of the research team trained on actigraphy procedures reviewed the record to achieve consensus scoring. Nocturnal sleep onset, sleep offset, and sleep minutes for the 7 nights before salivary melatonin collection were aggregated for weekday and weekend nights separately. Two weekend nights were available for all assessments except 6 (n = 2 in the younger cohort; n = 4 in the older cohort) in which only one weekend night was available. Five weekday nights were available for all assessments except 14; four nights were available in 13 assessments (n = 7 in the younger cohort; n = 6 in the older cohort) and three nights were available for one assessment in the younger cohort. Weekend and weekday differences for sleep onset and sleep offset were also computed.

DLMO phase, expressed in 24-hour clock time, was determined by linear interpolation across the time points before and after the melatonin concentration increased to and stayed above 4 pg/mL [29]. Phase angles were defined for each participant as the interval between DLMO phase and average weekday sleep onset (sleep onset time (decimal hours) minus DLMO phase (decimal hours)) and between DLMO phase and average weekday sleep offset ((sleep offset time (decimal hours) +24) – DLMO phase (decimal hours)).

Outcome measures by participant and assessment are available in Table S1.

### Statistical Analysis

The aim of this analysis was to describe developmental trajectories of sleep/wake timing, circadian timing, and phase angles of entrainment across adolescence. Therefore, statistical analyses for each outcome variable were carried out in three steps. In the first step, visual inspection of plots derived from non-parametric localized regression was used to examine the shapes of the developmental trajectories for each variable. In the second step, mixed model analyses were used to test for differences in variables across age in order to account for the multiple assessments per participant. Separate models were fit for the younger and older cohorts, a level-1 model testing for change in variables across chronological age using linear and quadratic trends and a level-2 model including a random effect for the intercept and linear trend, which allowed for variation among participants in these two parameters. Non-significant quadratic trends were dropped from final models. In the third step, two additional mixed models were run for each outcome variable in order to examine the influence of Tanner stage and sex on the developmental trajectories: 1) Tanner stage was dichotomized (<stage 3 vs. ≥ stage 3) and entered as a time-varying covariate at level-1 for the younger cohort; Tanner stage was not examined in the older cohort because all participants were≥ stage 3; 2) sex was entered in as a level-2 covariate and allowed to interact with the linear and quadratic trends. All analyses were run using SAS version 9.3 (SAS Institute Inc, Cary, NC).

## Results

In the younger cohort, 38 participants contributed 179 observations (mean per participant  = 3.98; range  = 1 to 6; n = 17 completed all 6 assessments) across the ages of 9 to 13 years. In the older cohort, 56 participants contributed 221 observations (mean per participant  = 4.74; range  = 1 to 6; n = 15 completed all 6 assessments) across the ages of 15 to 19 years. Demographic information for individuals at each age year is presented in Table 1.

**Table 1:** Demographics by individual at each age in the younger and older cohorts.

	Age (younger cohort)					Age (older cohort)			
	9	10	11	12	13	15	16	17	18+
	(*n* = 15)	(*n* = 35)	(*n* = 30)	(*n* = 24)	(*n* = 11)	(*n* = 43)	(*n* = 44)	(*n* = 38)	(*n* = 16)
Sex, n female	7	15	12	13	6	20	21	20	10
Race, n Caucasian	10	27	24	20	10	29	32	26	11
Tanner Stage, n									
1	8	19	6	1	0	-	-	-	-
2	5	10^a^	15^a^	10^a^	2	-	-	-	-
3	1	5	2^b^	6^c^	3^c^	-	-	-	-
4	-	1	2^d^	2	3^d^	7^d^	2	-	-
5	-	-	4^e^	5	2	34^f^	42^f^	38	16
Morningness/	41.0	41.6	38.9	38.3	35.2	35.7	35.6	34.8	33.9
Eveningness,	(4.5)	(5.3)	(6.2)	(5.1)	(2.8)	(5.5)	(6.3)	(6.5)	(8.4)
mean (SD)									
School Start Time,	8:20	8:28	8:19	8:03	7:59	7:46	7:49	7:56	-
mean (SD in mins)	(25)	(28)	(25)	(17)	(23)	(28)	(25)	(40)	

a Participants transitioned from Tanner 1 to 2 at 10 years (n = 5), 11 years (n = 6), and 12 years (n = 5).

b Participants transitioned from Tanner 1 to 3 at 11 years (n = 1).

c Participants transitioned from Tanner 2 to 3 at 12 years (n = 1) and 13 years (n = 1).

d Participants transitioned from Tanner 3 to 4 at 11 years (n = 2), 13 years (n = 1), and 15 years (n = 1).

e Participants transitioned from Tanner 3 to 5 at 11 years (n = 2).

f Participants transitioned from Tanner 4 to 5 at 15 years (n = 3) and 16 years (n = 1).

Notes: if more than one Morningness/Eveningness score was collected at each age, then the mean score was used; Tanner stage was unavailable for 1 participant at ages 9, 11, and 13 years, and for 2 participants at age 15 years.

### Developmental behavioral sleep trajectories

**Figure 1:**
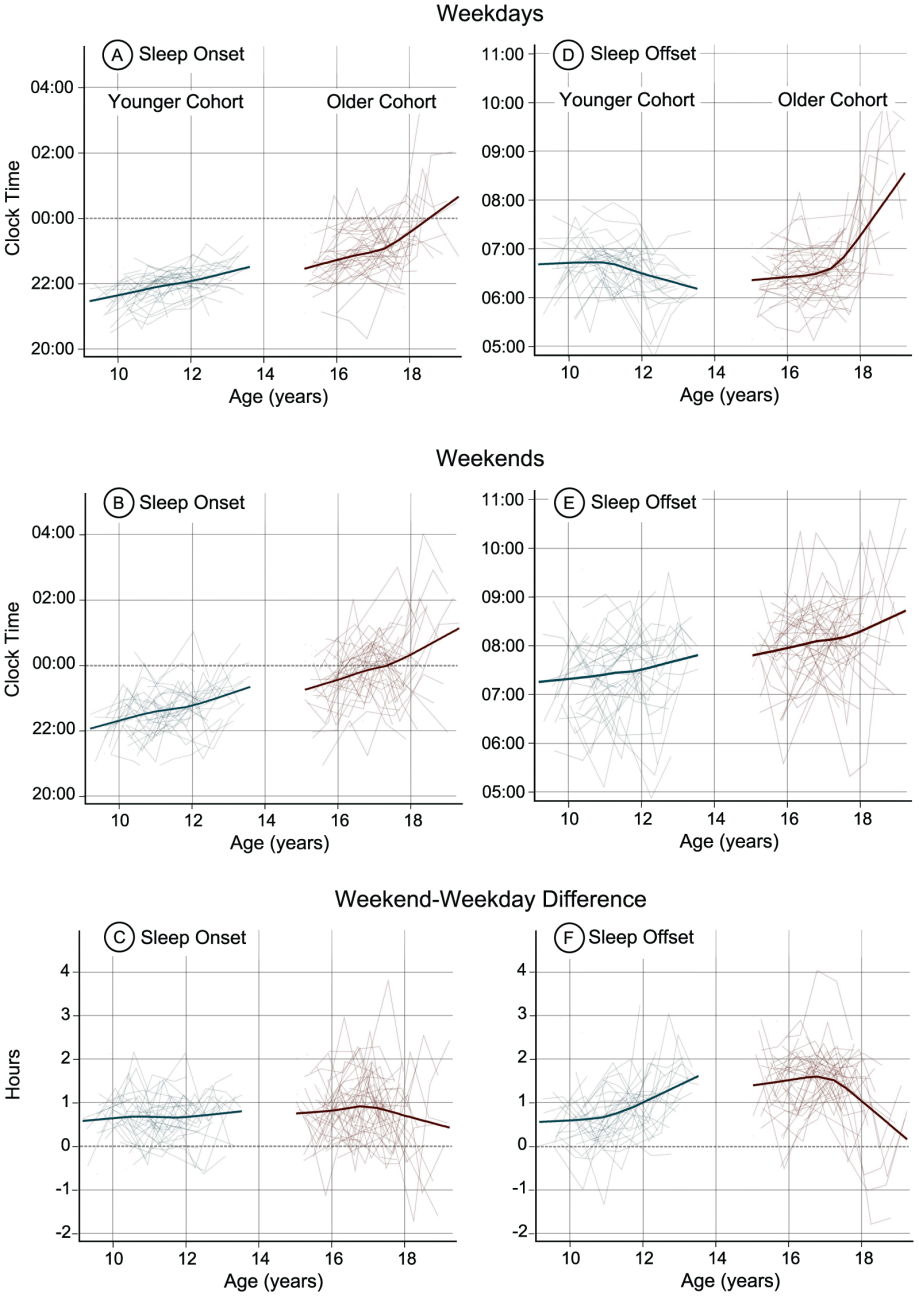
Modeled developmental trajectories (bold line) and individual trajectories (thin lines) for actigraphically estimated sleep onset and offset on weekdays (A and D) and weekends (B and E) in the proximal 7 days before DLMO phase was measured in both cohorts. Sleep onset and offset differences between weekends and weekdays (C and F) illustrate when participants slept earlier (<0) or later (>0) on weekends compared to weekdays. The younger cohort (9–13 years) is on the left and the older cohort (15–19 years) is on the right of each plot.

The mean values for each actigraphic sleep variable by age are presented in Table 2. The non-parametric developmental trajectories for actigraphic sleep variables by age are illustrated in Figure 1. Beginning with the younger cohort (left side of each plot in Figure 1), weekday and weekend sleep onset times (Figure 1 A and B) shifted later between the ages of 9 and 13 years, with significant linear trends for weekday (*F*(1,32) = 36.25, *p*<.01) and weekend (*F*(1,32) = 36.04, *p*<.01) sleep onset times. The weekend-weekday sleep onset difference (Figure 1C), however, did not show an age-related change in this younger cohort. Weekday sleep offset times shifted earlier in the younger cohort (Figure 1D) after age 11 (quadratic trend: *F*(1,100) = 6.60, *p* = .01). By contrast, weekend sleep offset times (Figure 1E) shifted later between 9 and 13 years, as shown by a significant linear trend (*F*(1,32) = 9.00, *p*<.01). The weekend-weekday differences for sleep offset (Figure 1F) increased from ages 9 to 13 years (linear trend: *F*(1,32) = 31.00, *p*<.01), which reflects sleep offset times shifting earlier on weekdays and later on weekends in the younger cohort.

**Table 2:** Means (SDs) for actigraphic sleep and circadian outcomes by age in the younger and older cohorts.

	Age (younger cohort)					Age (older cohort)			
	9	10	11	12	13	15	16	17	18+
	(*n* = 17)	(*n* = 56)	(*n* = 52)	(*n* = 38)	(*n* = 13)	(*n* = 55)	(*n* = 69)	(*n* = 64)	(*n* = 26)
Weekday sleep:									
Onset time	21:28	21:44	21:59	22:07	22:30	22:34	22:53	23:04	00:14
(mins)	(33)	(30)	(28)	(28)	(31)	(44)	(46)	(45)	(85)
Offset time	06:38	06:46	06:39	06:20	06:20	06:22	06:28	06:34	08:04
(mins)	(35)	(31)	(29)	(35)	(22)	(31)	(33)	(32)	(70)
Total sleep time, h	8.24	8.22	7.87	7.50	7.21	7.13	6.91	6.90	7.32
	(.85)	(.74)	(.68)	(.82)	(.70)	(.86)	(1.04)	(.98)	(1.37)
Weekend Sleep:									
Onset time	22:03	22:26	22:38	22:49	23:20	23:19	23:47	00:01	00:45
(mins)	(47)	(46)	(37)	(48)	(38)	(52)	(51)	(61)	(91)
Offset time	07:15	07:22	07:31	07:28	07:55	07:51	08:08	08:08	08:36
(mins)	(54)	(50)	(49)	(62)	(25)	(45)	(46)	(47)	(73)
Total sleep time, h	8.16	8.15	8.04	7.81	7.90	7.68	7.51	7.43	7.20
	(.98)	(.98)	(.93)	(1.05)	(.97)	(1.15)	(1.16)	(1.25)	(2.41)
Weekend-Weekday Difference:									
Sleep Onset, h	.58	.70	.65	.71	.83	.76	.90	.95	.51
	(.64)	(.56)	(.48)	(.57)	(.57)	(.72)	(.73)	(.79)	(1.13)
Sleep Offset, h	.62	.60	.87	1.12	1.58	1.47	1.67	1.58	.54
	(.78)	(.66)	(.67)	(.74)	(.33)	(.80)	(.67)	(.68)	(1.13)
DLMO phase	20:42	20:39	20:32	20:50	21:27	20:34	20:50	20:57	21:53
(mins)	(37)	(47)	(45)	(50)	(49)	(57)	(59)	(58)	(91)
Phase angle:									
DLMO to Weekday	.73	1.07	1.44	1.18	.99	2.05	2.06	2.13	2.17
Sleep Onset, h	(.90)	(.72)	(.78)	(1.06)	(.73)	(.66)	(1.04)	(.95)	(1.19)
DLMO to Weekday	9.88	10.11	10.12	9.44	8.87	9.86	9.64	9.66	10.07
Sleep Offset, h	(.78)	(.67)	(.69)	(1.19)	(.91)	(.84)	(.88)	(.94)	(1.21)

Notes: These data are from 94 participants (N = 38 in the younger cohort; N = 56 in the older cohort) who contributed on average 4.29 assessments range (1 to 6). Three observations at age 19 were included in the 18+ category.

Weekday and weekend sleep onset times also showed a delay in the older cohort (right side of each plot in Figure 1) between 15 and 19 years indicated by significant linear trends for weekday (*F*(1,39) = 17.55, *p*<.05) and weekend (*F*(1,39) = 13.69, *p*<.01) sleep onset time (Figure 1A and B). In contrast to the younger cohort, weekday sleep offset times delayed from 15 to 19 years, with a sharp delay after age 17 (Figure 1D). Significant linear (*F*(1,39) = 29.88, *p*<.01) and quadratic (*F*(1,123) = 35.81, *p*<.01) trends were seen for weekday sleep offset time. Weekend sleep offset times (Figure 1E) delayed from age 15 to 19 years in a linear fashion (*F*(1,39) = 10.26, *p*<.01). Unlike the younger cohort, weekend-weekday sleep offset timing differences decreased (Figure 1F), beginning from age 17 (linear trend: *F*(1,39) = 6.62, *p* = .01; quadratic trend: *F*(1,120) = 7.30, *p*<.01), reflecting the later weekday sleep offset times after age 17.

### Developmental circadian timing trajectories

**Figure 2:**
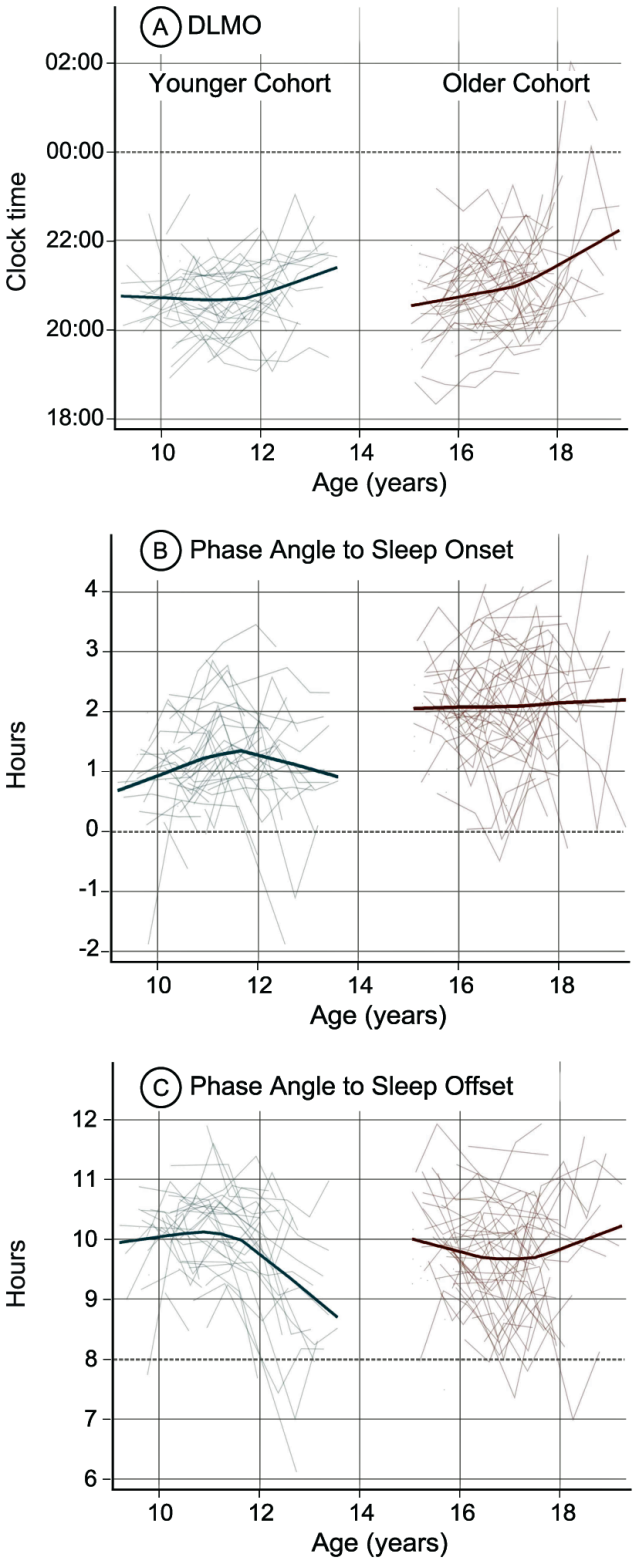
Modeled developmental trajectories (bold line) and individual trajectories (thin lines) for DLMO phase (A), DLMO phase angle to sleep onset (B), and DLMO phase angle to sleep offset (C). A negative DLMO phase angle to sleep onset indicates when the DLMO occurred after weekday sleep onset.

The mean values for each circadian outcome variable separated by age are shown in Table 2. Non-parametric developmental trajectories for DLMO phase, phase angle to weekday sleep onset, and phase angle to weekday sleep offset by age are illustrated in Figure 2.

For the younger cohort, DLMO phase (Figure 2A) began to show a delay after age 11, indicated by a significant quadratic trend (*F*(1,98) = 6.69, *p* = .01). Phase angle to sleep onset (Figure 2B) grew wider between ages 9 and 13 years, and this increase was most prominent between 9 and about 11.5 years. Significant linear (*F*(1,32) = 12.43, *p<*.01) and quadratic (*F*(1,91) = 13.36, *p*<.01) trends were seen for this measure. Phase angle to sleep offset time (Figure 2C) narrowed with age in the younger cohort after age 11 years. This pattern reflects the earlier timing of sleep offset on weekday mornings combined with DLMO delay after age 11. Phase angle to sleep offset time showed significant linear (*F*(1,32) = 7.21, *p* = .01) and quadratic (*F*(1,91) = 17.30, *p*<.01) trends.

Similar to the younger cohort, DLMO phase became later in the older cohort. The age-related delay was greater after age 17 (the inflection point in Figure 2A), indicated by a significant quadratic trend for DLMO phase (*F*(1,112) = 3.99, *p* = .05). Phase angle to sleep offset (Figure 2C) narrowed between about 15 and 17 years and then became wider again after age 17. Significant linear (*F*(1,39) = 7.18, *p* = .01) and quadratic (*F*(1,106) = 7.00, *p*<.01) trends were found for phase angle to sleep offset time. Phase angle to sleep onset time showed no age-related change in the older cohort (Figure 2B).

### Sex and Tanner stage

Developmental trajectories did not differ by sex for any variable except sleep onset on weekdays, which showed a more rapid delay shift with age in females than in males in the younger cohort (*F*(1,101) = 4.12, *p* = .05). Tanner stage added no statistically significant information above chronological age other than for sleep onset on weekdays in the younger group, in which Tanner stage ≥ 3 was associated with an approximate 12-minute later sleep onset on weekdays (*F*(1,100) = 5.11, *p* = .03).

## Discussion

This descriptive longitudinal analysis aimed to examine sleep/wake timing, circadian timing, and the temporal relations between measures of the internal circadian clock and the self-selected sleep patterns of younger and older adolescent cohorts that together spanned the second decade of life (9–19 years). The developmental trajectories observed in these two cohorts indicate that sleep/wake timing shifted later as youngsters aged, except in the case of weekday wake times, for which school schedules likely dictated the morning schedule. Overall, we also found an age-related delay of DLMO phase in both cohorts, with the largest (∼1 h) shift occurring between ages 11 and 13 years in the younger cohort and between 17 and 19 years in the older cohort. An age-related increase in phase angle to sleep onset was also seen in the younger cohort, especially between ages 9 and about 11.5 years. Participants in the older cohort fell asleep later relative to their melatonin rhythm compared to the younger cohort, as evidenced by a wider phase angle of DLMO to sleep onset. In addition, however, phase angle of DLMO to sleep onset remained consistent with age in the older cohort.

### Self-selected sleep/wake timing across adolescence

The age-related delay shift of weekday and weekend sleep onset and weekend sleep offset in both cohorts of the current study is consistent with previous work. Roenneberg and colleagues [10] for example, reported a significant age-related delay of about 2.5 hours in reported mid-sleep times of non-work/non-school (“free”) days in a cross-sectional analysis of individuals ages 10 to 20 years. Other previous cross-sectional survey findings showed a similar delayed pattern across the adolescent years [1], [21], though not as large as that reported by Roenneberg and colleagues. The younger cohort of the current study manifested a delay of sleep onset by about 1 hour (see Table 2). Two previous longitudinal studies showed a similar 1-hour parent-reported bedtime delay and actigraphic sleep onset delay during early adolescence, though clock times were descriptively earlier [2] or later [9] than the current sample. Such differences in clock times may be due to the way sleep timing was measured (parent report versus actigraphy) or to cultural factors. Wake-up time on weekends delayed by about 40 minutes with age in our younger cohort, and the discrepancy between weekend and weekday wake-up times increased from about 37 minutes at age 9 to about 50 minutes at age 13, on average (see Table 2). Laberge and colleagues [2] also reported such age-related changes to parent-reported wake-up times in their longitudinal assessment of adolescents in Canada, though the weekend wake time shift was descriptively longer (∼90 to 120 minutes, on average) by age 13.

The distinct changes in the developmental trajectories of sleep offset time observed in the current study (Figure 1D) likely reflect changes in the youngster's social structure, the most prominent of which are school start times. Previous work comparing sleep/wake schedules in response to differences in school start times showed that sleep onset times remain unchanged, but wake-up times shift earlier or later in tandem with the school schedules [36], [37]. In the current study, weekday sleep offset was about 20 minutes earlier at age 12 compared to the previous assessment ages. This is the age when American students transition from elementary to middle school, and the school schedule often begins earlier in the day, a pattern experienced by our participants (see Table 1). In the older cohort, weekday sleep offset remained consistent at about 6:20–6:30 am from ages 15 through 17 years, and a significant delay occurred when these youngsters reached 18 years. Most of the participants had completed high school by age 18, and no longer had the school schedule constraining sleep. A previous cross-sectional comparison [38] and longitudinal assessment [23] of sleep times across the transition from high school to college also showed a pattern of delayed wake-up times.

The consistent early weekday sleep offset times across 9 to 17 years, followed by a delay at age 18 and 19 years indicates that the school schedule may suppress a biologically-driven behavior to sleep later. This implication is bolstered by trajectories of later weekend sleep offset times in the two cohorts, as well as by the reduction of weekday-weekend sleep offset differences after age 17. Roenneberg and colleagues [39] reported that the degree to which weekend and weekday sleep timing differ increases over the second decade of life, and they relate the phenomenon to the construct of “social jetlag” (i.e., the degree to which social and biological clocks conflict). Larger degrees of social jetlag have been associated with reduced alertness and performance [40], [41], [42], [43], greater use of alcohol, nicotine and caffeine, and an increased risk for depression and obesity [39], [44], [45]. The current study's findings support a concern that exaggeration of social jetlag and potential associated health risks arise as adolescents' biological tendencies to delay are confronted by an early school bell.

### Circadian timing across adolescence

The circadian timing system, as indexed by DLMO phase, delayed with age in both cohorts; however, this age-related shift was non-linear, and the pattern of change was somewhat inconsistent with predictions based on weekday sleep/wake (dark/light) timing. Thus, for example, DLMO phase was stable across ages 9 to 11 years despite sleep onset time shifting later over this age range. If we apply the (presumed) properties of the phase response to light [46], [47], [48], [49], [50] such evening light exposure should produce a delay of circadian timing (i.e., later DLMO phase). Yet the younger cohort did not manifest this concomitant change, and we infer that DLMO phase was stabilized by morning rise-time consistency. One implication, therefore, is that the light phase response in the younger cohort is more sensitive in the morning than in the evening.

On the other hand, weekday wake-up times did not appear to influence DLMO phase in the same manner after age 11. DLMO phase shifted later after age 11 in the younger cohort and showed a delaying pattern between 15 and 17 years in the older cohort despite earlier sleep offset times on weekdays. Later sleep onset, and therefore exposure to ambient light later into the evening, may have driven a later DLMO phase at these ages. This finding is consistent with a previous study from this lab in which DLMO phase was later in adolescents across a transition to an earlier school start time accompanied by earlier wake-up times [51]. In combination, these findings suggest that the sensitivity of the circadian system to morning phase-resetting light exposure decreases and sensitivity to evening phase-delaying light increases with age during adolescent development. One study from juvenile mice showed a heightened phase delay response to evening light exposure [30], supporting the hypothesis for a developmental difference in the circadian clock's response to light.

### Phase angles of entrainment across adolescence

The phase angle from DLMO to sleep onset was wider in the older cohort compared to the younger cohort, meaning that the older adolescent group fell asleep at a later time on the melatonin rhythm. This finding is consistent with previous cross-sectional studies of adolescents [31]. In the context of findings from young children and adults, we note a pattern of larger phase angles to sleep onset associated with increasing age. Thus, for example, LeBourgeois and colleagues [52] recently reported a median DLMO phase to sleep onset interval of about 65 minutes in toddlers aged 30–36 months sleeping on their habitual parent-selected sleep schedules. Phase angles to sleep onset in our younger cohort and in a previously studied group of 9–12 year olds [31] was similar (∼1 h) to these toddlers. Our older cohort, however, had a phase angle to sleep onset average closer to 2 h, similar to that reported for adults on self-selected sleep schedules [53], [54], [55]. We suggest that the adult-like temporal alignment between sleep and the circadian timing system emerges during late adolescence and may be related to diminished parental involvement in setting bedtimes [1], [19], [21], [56], [57]. Although we did not measure parental involvement in this study, one might presume that release from parental-set bedtimes results in older adolescents choosing to go to bed later and thus falling asleep later with respect to their melatonin rhythm.

On the other hand, we suggest that developmental differences affecting the homeostatic sleep system can produce longer phase angles. Previous work illustrates that the dynamics of the homeostatic system are altered during adolescent development. While the decay of homeostatic sleep pressure across sleep does not change [27], [58], [59], the accumulation of sleep pressure across waking does. One cross-sectional study modeled the build-up of slow wave activity using sleep before and after 36 hours of sleep deprivation and found an increase in the time constant of the build-up in post-pubertal versus pre-pubertal teens [27]. In other words, mature adolescents accumulated sleep pressure at a slower rate across a waking interval compared to their younger peers. Longer sleep onset latency near bedtime in Tanner 5 adolescents compared to Tanner 1 adolescents following 14.5 and 16.5 hours awake provides further support for this developmental difference in sleep pressure [60].

**Figure 3:**
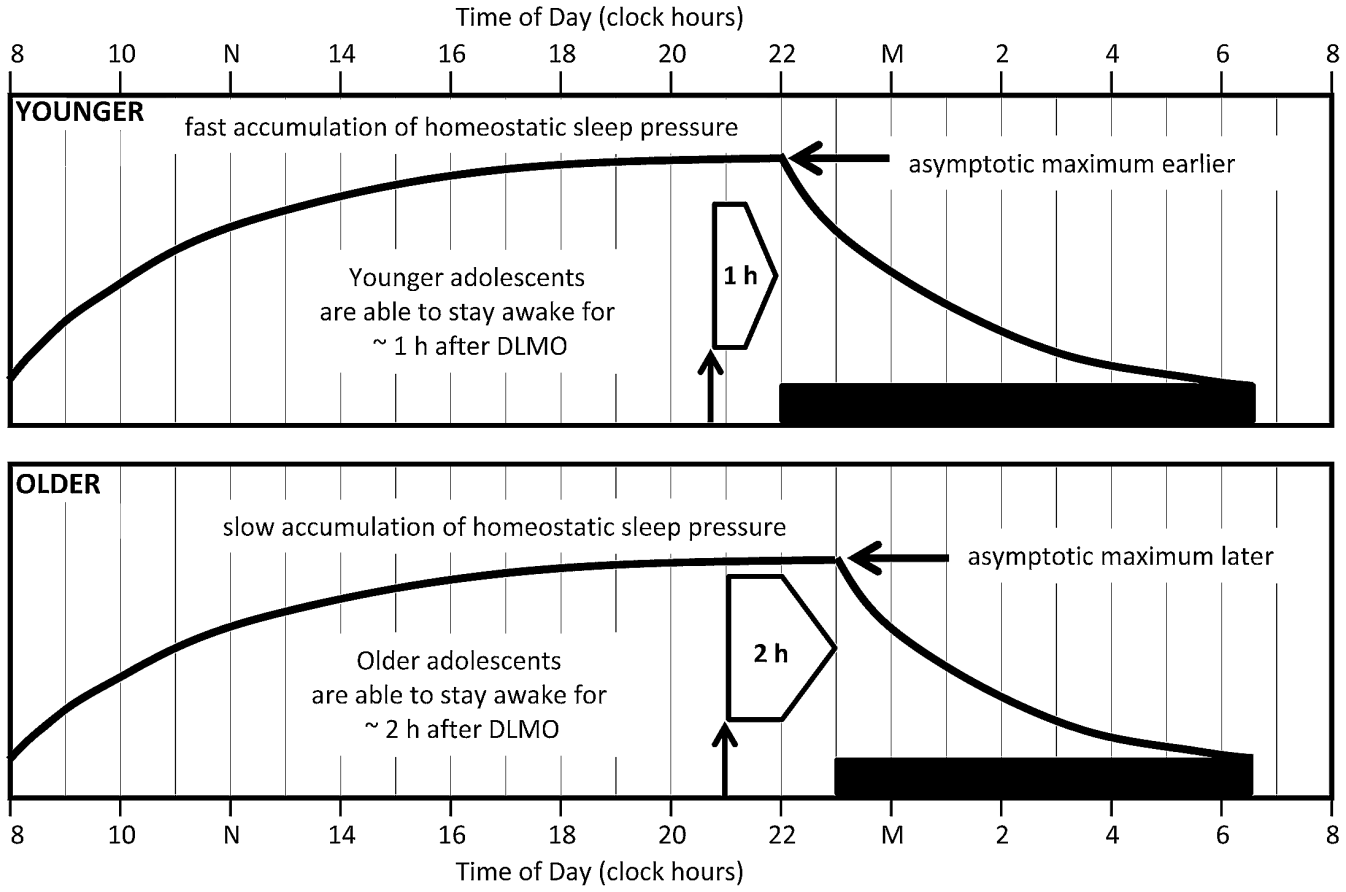
A proposed model to explain phase angle to sleep onset differences in the younger (top) and older (bottom) adolescent cohorts. Black horizontal bars illustrate average sleep times for each cohort (younger: 21:55–06:35; older: 23:02–06:40). Bold lines illustrate sleep pressure accumulation and dissipation functions predicted by the homeostatic sleep system. The upward facing arrow indicates the average DLMO phase for each age group (younger: 20:42; older: 20:54), and the right-facing block arrow shows the interval between DLMO phase and sleep onset (phase angle to sleep onset). Based on previous modeling work, [27] the saturating exponential function reaches it maximum more quickly and therefore at an earlier clock time in the younger cohort compared to the older cohort. We propose that the older adolescents are able to stay awake for a longer period of time (∼2 h) after DLMO phase compared to the younger adolescents (∼1 h) because of this developmental difference in homeostatic sleep pressure at the end of the waking day.

Figure 3 illustrates a model relating developmental changes in circadian timing and sleep/wake homeostasis to phase angle of DLMO and sleep onset that we propose to explain the phase angle differences between our age cohorts. Most assessments of the younger adolescents occurred at a developmental stage (Tanner stages 1 to 3) associated with achieving maximum sleep pressure earlier relative to rise time. On the other hand, the older adolescents were all late or post-pubertal (Tanner stages 4 and 5), a developmental stage at which sleep pressure builds more slowly across the day. Our younger cohort exhibited an average of 15 h 20 min between waking and falling asleep; whereas the waking day length was 16 h 22 min in the older cohort. We propose that the differences in accumulation of homeostatic sleep pressure may allow the older adolescent to stay awake for a longer period of time after their DLMO, thus extending the observed phase angle to sleep onset. These differences in day length can account for the longer phase angle in older adolescents (about 2 h) versus younger adolescents (about 1 h), as noted above.

### Study limitations and future directions

The longitudinal design and analytic approach of the current study allowed us to observe individual variability in developmental trajectories of sleep/wake behavior, DLMO phase, and phase angles of entrainment across early and late adolescence (see Figures 1 and 2). Developmental trajectories differed by sex and Tanner stage for weekday sleep onset in the younger cohort only. Therefore, other factors – perhaps genetic and/or environmental – likely influence an individual's pattern of change in sleep/wake and circadian timing across adolescence. Future work to examine these individual differences may inform our understanding of youngsters who could be at risk for developing circadian-based sleep complaints, such as a delayed sleep phase.

The current study had a number of strengths, including the longitudinal design and use of actigraphy and melatonin onset to describe developmental trajectories of sleep and circadian rhythms and the temporal alignment between these two processes across adolescence. The gap between the ages at the last assessment in the younger cohort and the first assessment in the older cohort is a significant limitation. Furthermore, the sample size limited our ability to examine sleep and circadian timing trajectories by sex. Recent data indicate sex differences in underlying circadian physiology and its temporal relationship to sleep [55], [61], [62]. Future longitudinal work examining sleep and circadian timing by sex may inform the developmental timing of sex differences in these parameters. Finally, the current analysis was intended to be descriptive. Examining the associations between the sleep and circadian timing trajectories and determining which may predict the other over time requires a different analytic approach (e.g., structural equation modeling) than the one used here. Future analyses are planned to examine associations between sleep timing and circadian timing trajectories, but that this analysis was beyond the scope of the current descriptive paper.

## Supporting Information

Table S1 **Outcome measures by participant and assessment.**
(XLS)
